# Protein and peptide confinement within metal–organic materials

**DOI:** 10.1039/d5cc01678a

**Published:** 2025-05-14

**Authors:** Jack D. Wright, Tongtong Zhang, Xiangyu Wang, Imogen A. Riddell

**Affiliations:** a Department of Chemistry, The University of Manchester Oxford Road Manchester M13 9PL UK imogen.riddell@manchester.ac.uk; b Manchester Institute of Biotechnology, University of Manchester 131 Princess Street Manchester M1 7DN UK

## Abstract

Metal–organic materials (MOMs), including both discrete metal–organic cages (MOCs) and metal–organic frameworks (MOFs), are emerging as promising materials for peptide and protein immobilisation. In particular, the ease of synthesis of MOMs alongside their well-defined and modular internal void spaces makes them appealing when considering routes to immobilise and stabilise peptides and proteins outside of biological environments whilst retaining their native activity. Here we review recent advances made in understanding the conformation of peptidic materials confined within MOMs and the enzymes@MOF constructs which show the best enzymatic performance. We highlight opportunities for further advancement in each of these areas and proposed that complementary approaches taken by the MOC and MOF communities might be fruitfully combined to advance our understanding and the development of peptide/protein@MOM applications.

## Abstract 2

Metal–organic materials (MOMs), including both discrete metal–organic cages (MOCs) and metal–organic frameworks (MOFs), are emerging as promising materials for peptide and protein immobilisation.

## Graphical abstract

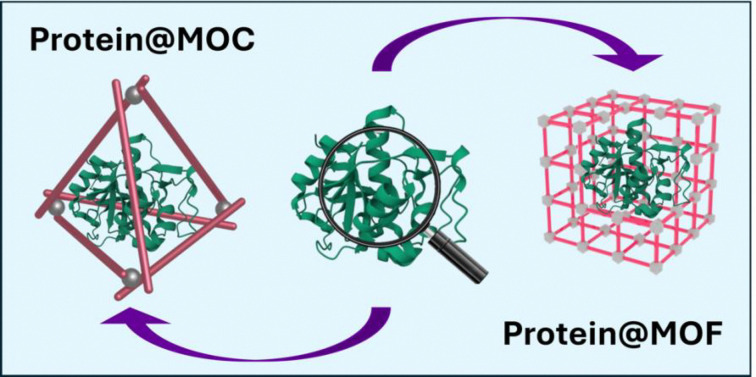

## 1. Introduction

Proteins and peptides often have intricate three-dimensional structures dictated by non-covalent intramolecular interactions.^1^ These non-covalent interactions are highly dependent on the chemical environment in which they are found and thus frequently preclude the translation of useful peptide-based processes beyond their native environment. Metal–organic materials, both discrete metal–organic cages (MOCs) and their polymeric counterparts, metal–organic frameworks (MOFs), have gained increasing attention as encapsulation matrices for proteins and peptides^2^ due to their atomically defined structures, modular self-assembly processes and well defined binding pockets which both enable characterisation of biomolecules within the metal–organic material (MOM) and can be designed to support the active conformation of the biomolecule.

Compared with MOFs, fewer examples of peptide binding within MOCs are reported. Key challenges for encapsulation of proteins and peptides in MOCs include limitations on the void space found inside the MOC, as well as solubility and stability challenges. In particular, examples of water-soluble MOCs that are stable and able to take up native peptides and proteins remain limited.^3–6^ The solution processability of MOCs does, however, provide characterisation opportunities when compared with protein@MOF constructs. The differential solubility of MOCs *versus* MOFs also presents opportunities for a wider range of applications.

Protein@MOFs can be synthesised through two routes, either by employing a biocompatible single-step synthesis,^7^ or alternatively in a two-step process whereby pre-formed MOFs bind peptides or proteins in a secondary step.^8^ The latter approach negates the need to synthesize the MOF under biologically friendly conditions. Regardless of the assembly process, the protein@MOF constructs generated ideally form a stable colloidal solution, with each particle typically being larger than a single protein@MOC complex.

This Feature Article summarises key examples of MOCs and MOFs which have been reported to encapsulate peptides or proteins. We focus on the benefits of peptide/protein encapsulation, looking at the approaches taken to characterise these composites and the opportunities presented by peptide/protein confinement. This is not intended to be a comprehensive review of all MOMs which have been employed for protein/peptide encapsulation as many excellent reviews, especially with regard to MOFs, have been reported in this area.^9^ Similarly, we do not discuss peptide/protein binding in organic materials^10^ nor do we discuss examples of peptide/protein binding on the surface of MOMs.^11^ Instead we focus on the limited examples of MOCs for peptide/protein encapsulation (Section 2) and the most common MOFs employed for peptide/protein encapsulation (Section 3.1). Sections 3.2 and 3.3 look at the conformational effects of peptide/protein encapsulation and recent advanced techniques employed to enable better characterisation of protein@MOF composites.

## 2. Metal organic cage hosts

### 2.1 Peptide binding within MOCs

A major impediment to studying the encapsulation of peptides within MOCs is the limited number of MOCs that are stable under biologically relevant conditions. In recent years however, there has been growing interest in water-soluble MOCs with the potential to act as drug delivery agents and biological sensors.^12^ However, despite these advances, relatively few examples of peptides or small proteins being encapsulated within MOCs have been reported in the literature to date and thus the potential for MOCs to stabilise specific peptide configurations is yet to be realised.

The first example of a MOC capable of encapsulating short oligopeptides was reported by Fujita and coworkers in 2005.^13^ This report employed a well-established Pd_6_L_4_ octahedral complex (1) which was generated from six equivalents of palladium ethylenediamine nitrate and four equivalents of 2,4,6-tri(4-pyridyl)-1,3,5-triazine. Water-soluble 1 has a large (474 Å^3^) hydrophobic void cavity^14^ which accommodates up to three amino acid residues, which may also be part of a longer peptide chain of up to six amino acids in total length.

**Fig. 1:**
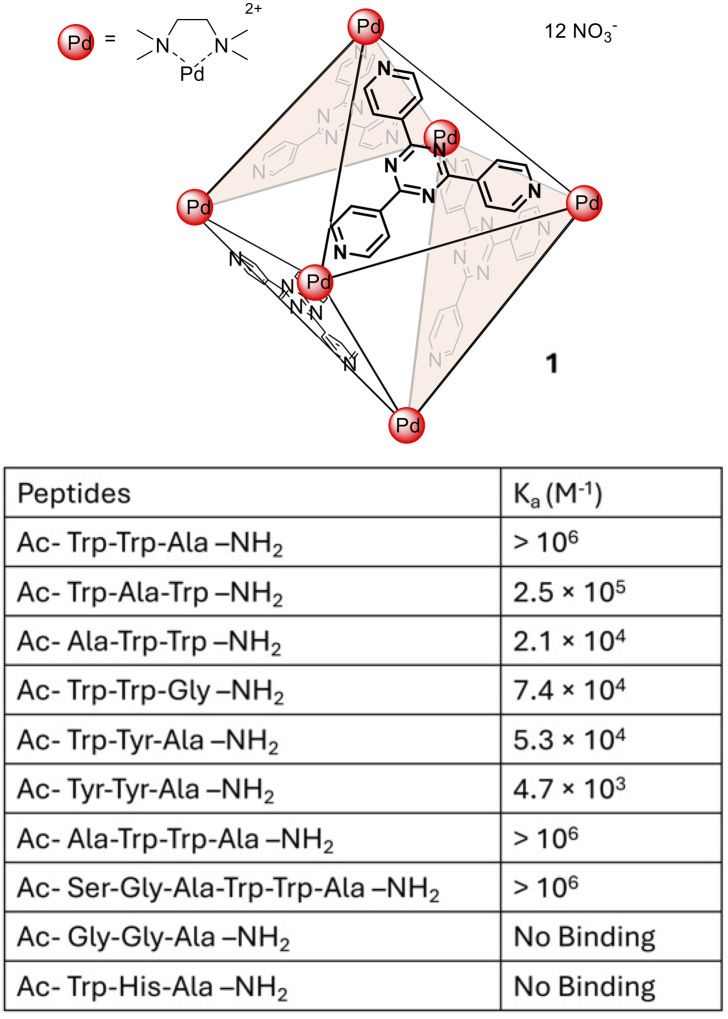
Water-soluble MOC 1, a Pd_6_L_4_ octahedron, and a tabulated list of peptides evaluated as guests and their association constants (*K*_a_).

Encapsulation of peptide sequences containing aromatic residues within 1 was supported by changes in the ^1^H NMR chemical shift of the peptide resonances. In contrast, when an oligopeptide without aromatic residues (Ac-Gly-Gly-Ala-NH_2_) and with a cationic residue (Ac-Trp-His-Ala-NH_2_) was investigated for host–guest binding it showed no changes in the chemical shift resonances consistent with a lack of binding. Further support for host–guest binding with appropriate sequences was provided through the observation of through-space NOE (nuclear Overhauser effect) interactions between 1 and the guest peptides. The proton resonances of 1 become more complex as the MOC desymmetrized upon peptide binding; therefore, ^1^H NMR titrations could not be employed to calculate binding constants. UV-Vis spectroscopy was therefore used and revealed sequence selective binding behaviour whereupon oligopeptides containing Trp-Trp-Ala sequences showed the largest association constants (*K*_a_, Fig. 1). The authors hypothesize that the preference for the Trp-Trp-Ala sequence is due to electron donation from the electron-rich indole rings of tryptophan to electron-deficient ligands of the MOC. With longer peptide sequences only the Trp-Trp-Ala resonances adjacent to the N-terminus show a significant upfield shift upon equilibration with 1 suggesting that this tripeptide motif is most favoured.

Overall analysis of the association constants (*K*_a_) for several oligopeptide sequences (Fig. 1) was consistent with the main driving force for encapsulation being π–π stacking between the aromatic ligands on the host and the hydrophobic peptide residues, namely tryptophan. The order and orientation of the amino acids within the peptide sequences was also shown to impact the strength of binding with greater *K*_a_ values reported for peptides containing two adjacent tryptophan residues.

**Fig. 2:**
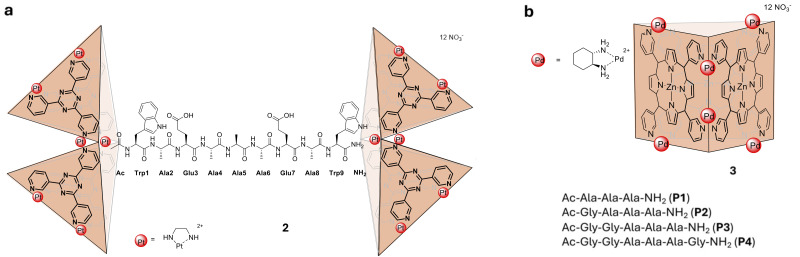
(a) The 2 : 1 MOC : peptide complex formed with MOC 2, a Pt_6_L_4_ bowl and peptides up to nine amino acids in length; (b) the zinc porphyrin MOC 3 reported to bind short peptides P1–P4 inducing defined peptide conformations.

**Fig. 3:**
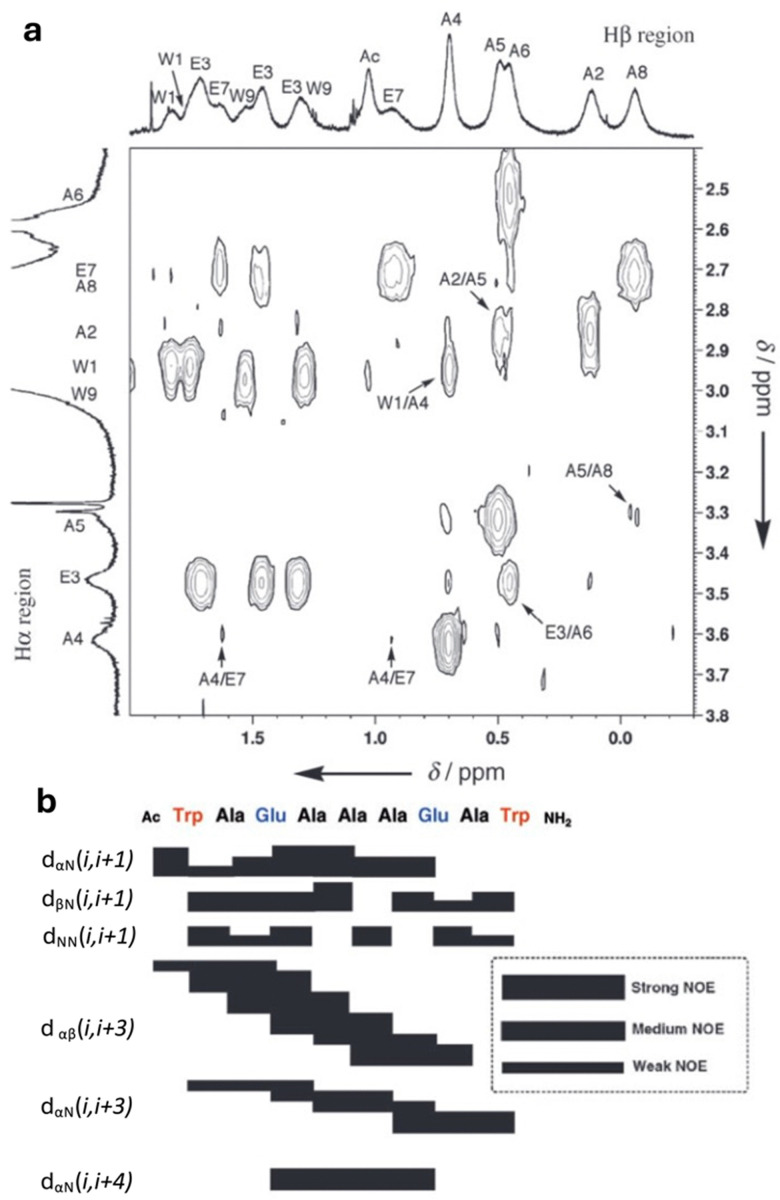
(a) Selected NOESY spectrum for the assignment of bound peptide (600 MHz, H_2_O/D_2_O = 9/1, 27 °C, 100 mM phosphate buffer, pH 6.8), [2] = 10 mM, [peptide] = 2.5 mM. Proton resonances labelled with single letter amino acid code and residue number corresponding to peptide in Fig. 2a; (b) NOE correlations for peptide⊂2. Reproduced with permission from ref. 16.

Fujita and coworkers further expanded their studies into peptide binding MOCs using a Pt_6_L_4_ bowl-like structure, 2, self-assembled from 2,4,6-tri(3-pyridyl)-1,3,5-triazine and platinum ethylenediamine nitrate. MOC 2 was shown to bind peptides up to nine amino acid residues in length (Fig. 2a).^15^ The sequences were shown to bind in both a 1 : 1 and 2 : 1 host : guest ratio with computational models and NMR data supporting the oligopeptides being bound in an α-helical conformation when encapsulated.^16^ Upfield shifts in the peptide resonances upon encapsulation within 2 and nuclear Overhauser effect (NOE) correlations supported an α-helical conformation of the peptide and indicated its positioning within 2. In particular, the observation of *d*_*αβ*_ (*i*,*i* + 3), *d*_*αN*_(*i*,*i* + 3) and *d*_*αN*_(*i*,*i* + 4) correlations (Fig. 3) provide strong support for an α-helix conformation.

Again UV-vis spectroscopy was used to obtain host–guest binding affinities. Two control peptides were used, one with a Trp1/Ala1 mutation (PepNterminus) and another with a Trp9/Ala9 mutation (PepCterminus), to investigate which tryptophan residue bound more strongly. Consistent with NMR data the PepCterminus showed a 5-fold increase in *K*_a_ (8.6 × 10^4^ M^−1^) compared with a *K*_a_ 1.6 × 10^4^ M^−1^for the PepNterminus.

Similarly, Fujita and coworkers demonstrated binding of short peptides within a zinc porphyrin MOC (3; Fig. 2b).^5^ The peptide sequences chosen adopt random coil conformations in water but when bound within 3 the peptides were shown to adopt mixed β-turn (3_10_)/α-helix (4_13_) conformations with the length of the peptide determining the conformations. For tripeptide P1 a 3_10_ helix conformation was observed; however, as the peptide was extended to four and more aromatic residues (P2, P3 and P4) a mixed 3_10_/4_13_ helical structure was observed *via* single crystal X-ray diffraction.

The lack of disorder in the peptide conformations observed by single-crystal X-ray diffraction of each of the peptides bound within 3 contrasted with the disorder observed in solution state studies. A crystal structure of P1⊂3 was grown through the slow evaporation of a solution of the host–guest complex from water, and solution and refinement revealed that the host–guest structure crystallised in the chiral space group *P*222. Single crystal data of P1⊂3 revealed consistent hydrogen bonding distances to those obtained through NOESY measurements supporting the solution and solid-state conformational similarity of P1⊂3. Moreover, molecular dynamics simulations of P2–P4 produced similar helical conformations to those observed in the respective host–guest crystal structures leading the authors to propose that encapsulation enables the peptides to exhibit their innate conformational preference rather than an artificial conformation determined by the confines of 3.

**Fig. 4:**
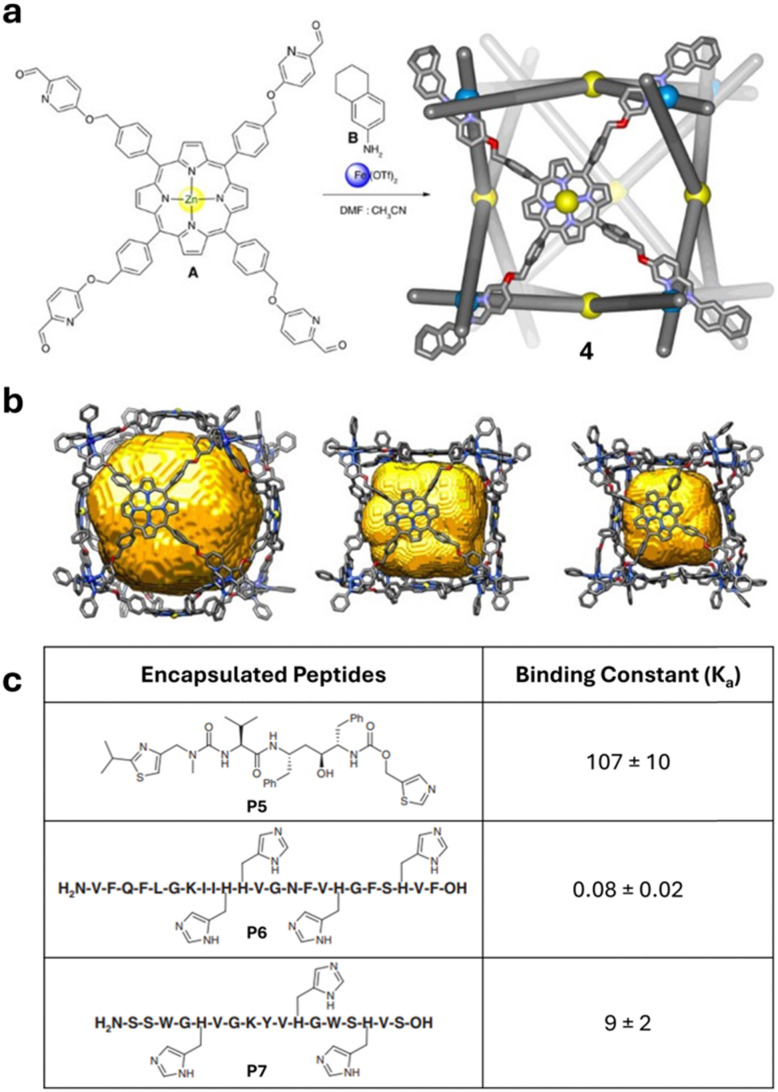
(a) Self-assembly of 4, a cubic MOC with one zinc porphyrin per face; (b) MM2 energy minimised models of 4 showing the structural flexibility and variable void volumes from 10 083 to 3143 Å^3^; (c) peptides P5–P7 found to bind within 4 and their binding constants. Reproduced with permission from ref. 6.

Despite the early successful demonstration of peptide binding within MOCs, no further work was carried out in the field until 2017 when Nitschke and coworkers reported an Fe_8_L_6_ cube (4) comprised of iron(ii) trispyridylimine vertices (Fig. 4).^6^ MOC 4 was flexible with a variable internal cavity volume and each face of the cube incorporated a zinc-porphyrin capable of coordinating with histidine moieties, a biologically recognised binding motif.^17^ MOC 4 was shown to bind and stabilise peptides up to 23-residues in length but due to solubility limitations this encapsulation needed to be performed in a 1 : 1 water : acetonitrile mixture. Encapsulation studies targeted the biologically relevant molecules ritonavir (P5), an antiretroviral medication for HIV/AIDS,^18^ containing two thiazole moieties, and clavanin A (P6), a 23-residue oligopeptide that is widely used as an antibiotic,^19^ as well as an abiotic peptide (P7) with one fewer histidine residue than clavanin A. Dissociation constants (*K*_d_) were determined using UV-vis spectroscopy. Amongst the guests investigated ritonavir showed the strongest binding with a *K*_d_ of 107 μM with three ritonavir molecules bound in every host. Both P6 and P7 were found to bind in a 1 : 2 H : G ratio with 4; however clavanin A had a 100-fold weaker *K*_d_ than its abiotic analogue P7 (*K*_d_ = 80 nM P6*vs.* 9 μM P7). The decrease in the dissociation constant of P6 with respect to P7 highlights the importance of properties beyond residue number and positioning as P7 is more hydrophilic than P6 and might have been predicted to more readily dissociate from 4.

Smaller histidine containing guests, as well as non-histidine containing guests were also studied with regard to their binding within 4. As expected, oligopeptide sequences that did not contain histidine residues showed no evidence of binding, supporting the essential role of the zinc porphyrin in this host–guest interaction. Meanwhile, all histidine containing molecules investigated bound regardless of size.

Beyond measurement of the guests’ affinity to 4, the potential to protect peptide guests from hydrolysis upon encapsulation was also studied. These studies utilised P7, which had greater solubility than either ritonavir or P6. Following addition of trypsin (0.6 eq.) to P7⊂4 only 9% of the peptide was observed to be cleaved as assessed by quantitative HPLC analysis. In contrast, when 0.3 equivalents of trypsin were added under otherwise identical conditions to a solution of P7 in the absence of 4, 76% of the peptide was cleaved. Control experiments with zinc(ii) tetrakis(4-sulfonatophenyl)porphyrin indicated that peptide interaction with the porphyrin was insufficient to protect the oligopeptide from trypsin, indicating that encapsulation is key to preventing peptide hydrolysis.

In 2024, Fujita and coworkers demonstrated that an expanded version of MOC 1 could be accessed through solid state synthesis with an appropriate template molecule.^14^ The large M_9_L_6_ complex (5) had an internal cavity >1500 Å^3^, representing a greater than three-fold increase compared with 1. MOC 5 is capable of encapsulating medium sized molecules (up to ∼1600 molecular weight), which bridge the gap between small molecules and large biomolecules such as proteins and DNA/RNA. Due to the water-soluble nature of 5 and its intermediates, the self-assembly reaction could be followed *via*^1^H NMR spectroscopy and mass spectrometry, and host–guest studies with 5 indicate that it was capable of encapsulating large guests. Rifampicin (P8), an antibiotic with a molecular weight of 822.94, was found to bind in a 1 : 2 host : guest ratio with 5. The macrocyclic peptide, daptomycin (P9) (*M*_r_ = 1619.70) also bound in a 1 : 2 host : guest ratio and strong cooperative binding was observed through UV-vis titrations. Association constants for P9⊂5 of *K*_a_1 = (4.8 ± 1.2) × 10^4^ M^−1^ for the first complexation (1 : 1) and *K*_a_2 = (2.6 ± 0.2) × 10^5^ M^−1^ for the second complexation (1 : 2) were reported. Binding was supported through the hydrophobic decanoate, indole, and aniline moieties of the side chains of P9, which were proposed to form a hydrophobic dimer within the void cavity of 5. The authors posit that novel methods of expanding cage sizes, such as those presented, often lead to significant expansion of the application scope of MOCs.

### 2.2 Protein binding within MOCs

Moving from peptide to protein guests, Fujita and coworkers reported the encapsulation of ubiquitin A within a giant Pd_12_L_24_ MOC (6) in 2012.^3^ The study primarily utilised DMSO as a solvent, rather than water, but it represents an important milestone towards aqueous protein encapsulation. Ubiquitin A is a 76-residue protein which was chosen due to its small size and the potential to be covalently linked with one of the organic linkers that make up the MOC. Following direct attachment of ubiquitin to the bidentate ligand (L1), self-assembly of 6 was performed using a 12 : 1 : 23 metal : ubiquitin-functionalised-L1 : L1 ratio. This self-assembly approach resulted in the formation of a well-defined coordination cage with one ubiquitin protein per cavity. Ubiquitin⊂6 was analysed using a combination of NMR spectroscopy, single crystal X-ray diffraction and analytical ultracentrifugation (AUC).

Due to the high molecular weight of the host : guest complex AUC provided a convenient approach to the separation of ubiquitin-functionalised ligand and ubiquitin⊂6. Moreover, sedimentation equilibrium analysis enabled the experimental estimation of the molecular weights of ubiquitin⊂6 and ubiquitin-functionalised-L1 as 26 300 and 16 300, respectively. This was in good agreement with their theoretical values of 25 300 and 16 700.

Diffusion-ordered spectroscopy (DOSY) NMR performed on purified solutions of 6 and ubiquitin⊂6 indicated the proton resonances corresponding to bound ubiquitin had the same diffusion coefficient (*D*) as 6 (*D* = 5.7 × 10^−11^ m^2^ s^−1^, log *D* = −10.24), while individually free ubiquitin and 6 have diffusion coefficients of *D* = 1.0 × 10^−10^ m^2^ s^−1^, log *D* = −9.98 and *D* = 7.8 × 10^−11^ m^2^ s^−1^, log *D* = −10.11, respectively. This observation supports the internal binding of ubiquitin within 6. Finally, single crystals suitable for X-ray diffraction were obtained by slow diffusion of isopropyl acetate into a dimethyl sulfoxide solution of 6. Structure solution and refinement using the maximum-entropy method (MEM) enabled the electron density of the protein to be mapped to ubiquitin⊂6 providing further support for binding of the ubiquitin within the central cavity of 6.

Building on the initial studies showing ubiquitin inside of a Pd_12_L_24_ MOC Fujita and coworkers next looked at the dynamic covalent attachment of cutinase-like enzyme (CLE) within a MOC.^4^ CLE is a bacterial enzyme known to degrade plastic,^20^ and it was chosen as a model guest protein as its size aligns well with the interior cavity of 6 and it exhibits good inherent stability under the reaction conditions necessary for self-assembly of the Pd_12_L_24_ MOC. As with previous examples the limited MOC solubility in aqueous media necessitated a 1 : 1 buffered aqueous : acetonitrile solvent be used for all studies of CLE⊂6; however, the inclusion of water is a positive step towards fully aqueous conditions. DOSY NMR spectra as well as the modified enzyme activity provided compelling evidence for CLE encapsulation. Control studies confirm that in a 1 : 1 buffered aqueous: acetonitrile solution the activity of CLE was not impeded, as observed by tracking products from the enzymatic hydrolysis of 4-nitrophenyl laurate.^21^ At higher ratios of organic solvent the CLE did, however, display diminished activity, by up to 20% of the original efficacy. The original activity could, however, readily be restored by returning the host–guest complex to the original reaction conditions. In addition to increased stability to organic solvents, encapsulation within 6 also provided thermal protection to CLE. Capillary-differential scanning calorimetry (DSC) assessed the thermostability of CLE⊂6 and found that the melting point of CLE increased to 130 °C when encapsulated, and this was raised from 40 to 50 °C in the native CLE.

**Fig. 5:**
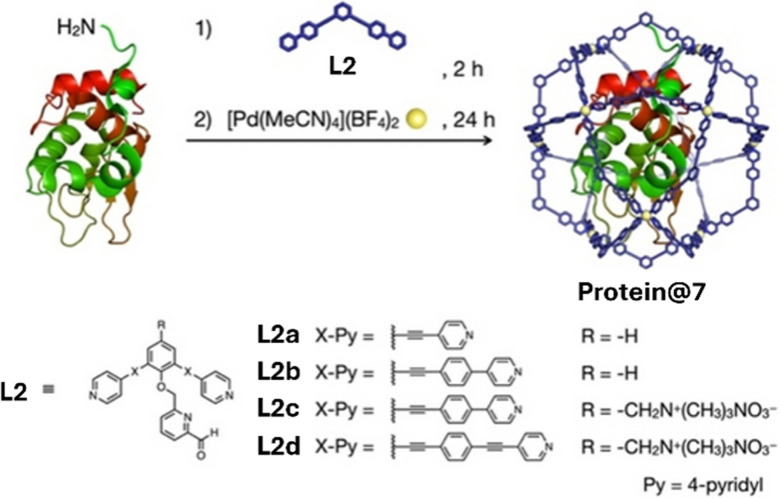
Encapsulation of a protein within a M_12_L_24_ MOC (7) and the range of linkers (L2a–d) employed to generate isostructural cages with a range of internal void cavities. Reproduced with permission from ref. 22.

Utilising 7, which has the same overall structure as 6 but includes a pyridine carboxaldehyde linker (L2; Fig. 5) in place of the maleimide linker (L1), protein encapsulation has been broadened to include a further 15 types of proteins with varying properties (*e.g.* hydrophobicity, surface charge and length).^22^ Evidence for unimolecular protein⊂7 encapsulation was determined by AUC and DOSY NMR spectroscopy. Proteins encapsulated range in size from insulin (51 amino acids) to thermolysin (316 amino acids) and were stable in 1 : 1 H_2_O : MeCN solutions. When encapsulated inside 7 trypsin demonstrated increased stability against heat and organic solvents (60 °C and up to 90% MeCN) providing further support that MOCs show great potential in stabilising proteins in harsh conditions.

Despite the limited examples in the literature of peptide and protein encapsulation within MOCs, the opportunities for these systems are clear. Encapsulation provides the potential to (i) enforce peptide conformations not found in bulk media; (ii) stabilise active enzymatic conformations under atypical conditions, including at elevated temperatures and in organic solvents, and (iii) provide amino acid sequence-based recognition of peptides.

## 3. Metal organic framework hosts

### 3.1 MOF based strategies for enzyme encapsulation

In contrast to MOC studies, researchers working on protein binding in MOFs initially focussed on the preservation of enzymatic activity following encapsulation and only recent reports have started to probe conformation effects of encapsulation (see Sections 3.2 and 3.3). A wide variety of MOFs have thus been investigated for protein binding, including 2D and 3D frameworks synthesised through both post-synthetic and *in situ* assembly strategies. 2D MOFs defined as layered structures have metal ligand connectivity in two dimensions, and interlayer channels which provide opportunities for protein incorporation and substrate and product transport between enzyme active sites. The lower dimensionality of 2D MOFs does, however, also presents opportunities for protein leaching from the framework. In contrast, 3D MOFs are connected in all three dimensions: incorporated proteins can thus only be lost by breakdown of the framework, or through the MOF pores. Similarly, substrates and products must be transported through continuous channels in the MOF.^23^ Post-synthetic approaches enable use of MOFs that require synthetic conditions incompatible with enzymes (elevated temperatures, pH range or organic solvents); they are, however, limited by the pore size of the MOF, and the requirement for the enzyme to be able to fit through the pore without becoming denatured. Moreover, as the enzyme must be able to pass through the MOF pore it is also more likely to be lost through leaching than an enzyme encapsulated *via* an *in situ* enzyme@MOF synthetic approach. Two *in situ* enzyme@MOF synthetic strategies have been classified in the literature: coprecipitation and biomimetic crystallisation, also known as biomimetic mineralisation. These approaches differ with proteins being passively incorporated into the MOF structure in coprecipitation reactions and contrastingly acting as essential MOF nucleation agents in biomimetic crystallisation reactions. Moreover, coprecipitation reactions employ coprecipitation agents, often polymers, in addition to the metal ions and organic ligands required to make up the MOF framework. The coprecipitation agent's role is to protect the enzyme during the self-assembly process and it typically supports the formation of amorphous rather than crystalline materials. Biomimetic crystallisation reactions include only metal ions, organic linkers and enzyme, and generate crystalline matrices in the presence of a suitable enzyme or biomolecule. Both approaches require the individual reaction components to have good solubility enabling the precipitated material to be isolated following completion of the reaction. The most commonly employed MOFs for protein encapsulation, including imidazole- and carboxylate-linked systems, are reviewed below.

#### 3.1.1 Imidazole-based MOFs

The zeolitic imidazolate frameworks (ZIFs) are a family of imidazole-based MOFs that include ZIF-8, ZIF-90, MAF-7, and ZIF-67 amongst others.^24^ The good aqueous solubility and biocompatibility of imidazole and its derivatives have resulted in the extensive investigation of ZIFs as biomimetic crystallisation materials.

**Fig. 6:**
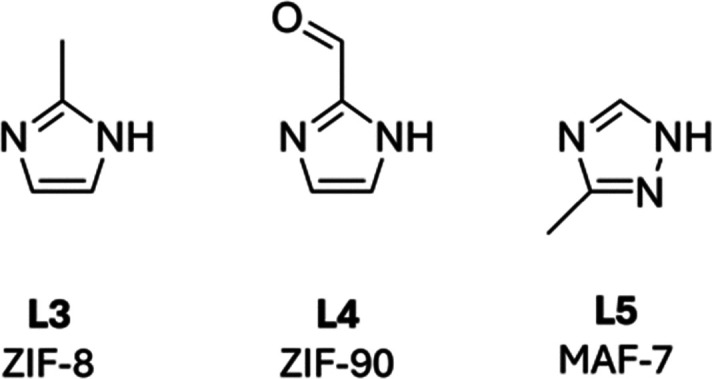
Imidazole linkers most commonly employed in enzyme@MOF *in situ* synthesis procedures including coprecipitation and biomimetic crystallisation approaches. ZIF-8 incorporates L3, ZIF-90 incorporates L4 and MAF-7 incorporates L5.

ZIF-8 was the first and is the most extensively investigated MOF employed in the one-pot, *in situ* synthesis of enzyme@MOFs. Critical to its success is that both precursors, the zinc(ii) salt and L3 (Fig. 6), have good aqueous solubility and do not denature enzymes under the conditions of a biomimetic crystallisation process. The incorporation of a suitable protein within the crystallisation reaction of ZIF-8 is essential, with proteins with negative surface charges shown to promote nucleation and crystal growth.^25^ The inclusion of a protein to promote MOF crystallisation has also been shown to direct the topology of the crystal and a range of topologies have been reported for protein@ZIF composites amongst which sodalite (SOD; Zn(L3)_2_) and ZIF-C (Zn_2_(L3)_2_CO_3_) exhibit the most common topologies.^26^ Both of these topologies have sufficient pore diameters to support the uptake of substrates and egress of products from encapsulated enzyme active sites. Moreover, the stable ZIF-8 structure has been shown to preserve the natural catalytic behaviour of many enzymes under conditions, which would otherwise denature the enzyme.^27^

In comparison to biomimetic crystallisation, coprecipitation has been investigated. In 2016 Doonan, Falcaro and coworkers immobilised polyvinylpyrrolidone (PVP)-modified urease and native urease in ZIF-8.^28^ For urease@ZIF-8 prepared by both biomimetic crystallisation and coprecipitation there was a significant improvement in the catalytic activity compared with that observed for the native enzyme. Biomineralised urease@ZIF-8 did, however, show better stability following thermal treatment over urease@ZIF-8 prepared *via* the coprecipitation method. The role of PVP was thus proposed to be protection of the enzyme from deactivation during the co-precipitation step when performed in alcoholic solvents. When the coprecipitation reaction was performed in aqueous solution PVP was not found to enhance the urease enzymatic activity or stability.^28^

The MOF particle dimensions and crystallinity^29^ have also been found to vary based on the synthetic conditions employed for the formation of enzymes@MOFs. Variation in the precursor concentration and reaction stoichiometry,^30^ as well as the choice of enzyme^31^ to be incorporated have given rise to hexagonal prisms and nanoflower morphologies amongst others, both of which show higher activity than the standard rhombic dodecahedral morphology of ZIF-8. A study by Doonan and coworkers has also shown that catalytic activity is highly sensitive to changes in the particle size, with improved catalytic activity attributed to the increased surface area of the particles and reduced diffusion limitations associated with the substrate accessing the enzyme active site.^32^ Additionally, inclusion of a peptide modulator, γ-poly-l-glutamic acid, has been shown to alter the structure and properties of enzyme@MOF composites.^23^

Zha and coworkers also reported the strengthened pH and thermal stability of lipase@ZIF-8 which they attributed to bonding interactions including hydrogen bonding and hydrophobic or electrostatic interactions between the lipase and ZIF-8.^33^

ZIF-90 and MAF-7 are zinc(ii) based 3D MOFs. ZIF-90 utilises 2-imidazole carboxaldehyde (L4)^34^ in place of L3 as the organic ligand and has a pore aperture of 3.5 Å and an internal pore diameter of 11.2 Å. MAF-7 incorporates 3-methyl-1,2,4-triazole (L5) as the organic linkers and forms a MOF with a pore diameter of 11.2 Å and a pore aperture of 3.4 Å. Unlike ZIF-8, both ZIF-90 and MAF-7 have a hydrophilic surface and channel interface which could hypothetically promote the transport of substrates in aqueous solvents. Studies by Liang and coworkers have, however, found that when catalase or urease was immobilised within hydrophilic ZIF-90 or MAF-7, the enzyme@MOFs exhibited retained activity, but showed no enhanced enzymatic behaviour over the native enzyme. In contrast, when catalase or urease was immobilised in ZIF-8 it led to a loss of activity compared with the native enzyme.^35^ Similarly, others have demonstrated that enzymes immobilised into ZIF-90 displayed retained activity compared to free enzymes. Although the enzyme@ZIF-90 MOFs did not acquire the desired enhanced activity, their stability was greatly improved after immobilisation.^36^

In addition to variation of the organic linker, the metal ion employed in imidazole-based MOFs has also been varied. ZIF-67 is a cobalt(ii)-based MOF which employs L3 as the organic linker. As with zinc-based systems, enzyme@ZIF-67 constructs show enhanced stability of the incorporated enzyme and can be recycled following a simple separation of the heterogeneous enzyme@MOF particles.^37^

#### 3.1.2 Carboxylate-based MOFs

Following the seminal work demonstrating the one-pot enzyme immobilisation within ZIFs, many carboxylate-based MOFs have been explored for enzyme encapsulation including those from the Materials of Institute Lavoisier frameworks (MILs),^38^ Hong Kong University of Science and Technology framework (HKUST-1) and University of Oslo-66 (UiO-66) families. Deprotonation of the ligands in the precursor solutions often facilitates solubilisation of the ligands within aqueous solution. Thermal and/or acidic treatment of the metal salts may also be carried out to pre-assemble the metal node prior to MOF formation,^39,40^ and modulators^41^ may be included within reaction mixture to promote the formation of a single crystalline MOF.

**Fig. 7:**
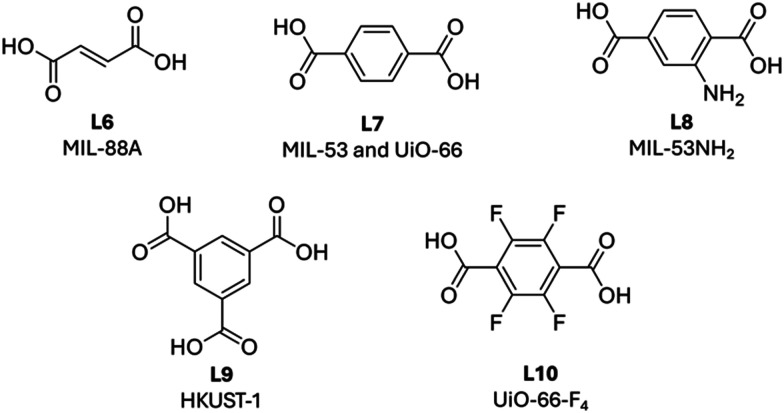
Carboxylic acid linkers employed in the biomimetic crystallisation of MIL-88A (L6), MIL-53 and UiO-66 (L7), MIL-53NH_2_ (L8), HKUST-1 (L9) and UiO-66-F_4_ (L10).

The MILs most commonly employed for *in situ* enzyme@MOF encapsulation are MIL-88A, MIL-53 and MIL-53NH_2_ consisting of dicarboxylic ligands L6–L8 (Fig. 7) and iron(iii) or aluminium(iii) ions. Early experiments investigating bovine serum albumin (BSA) immobilisation within MIL-88A revealed the PXRD pattern of BSA@MIL-88A corresponded with the standard MIL-88A powder X-ray diffraction (PXRD) pattern indicating no loss in the long range order.^7^ Later reports looked at immobilisation of a range of enzymes within MIL-88A(Fe) including phytase, xylanase, amylase, mannanase, and glucanase, and the controlled release of these enzymes from the MOF, which could be modulated by the incorporation of MOF defects.^42^ The utilisation of strong Lewis acidic cations (Fe^3+^ or Al^3+^) in MILs enables incorporation of a wider range of proteins than can be achieved with zinc(ii)-based MOFs. Lewis acids, such as iron(iii), have been found to interact with protein surfaces, regardless of their electrostatic properties, and thus support MOF nucleation.^43^ More recently, MILs incorporating aromatic organic ligands (L7 and L8) have attracted attention with Sánchez-Sánchez and coworkers demonstrating that the presence of lipase CalB could affect the topology of the MIL-53NH_2_(Al). At the same time, lipase CalB with a higher isoelectric point effectively accelerated the mineralisation rate of enzyme@MIL-53NH_2_(Al) when compared to β-glucosidase which has a lower isoelectric point.^44,45^ Ovalbumin, laccase and HRP have also been immobilised into MIL-53NH_2_(Al and Fe) and employed for novel applications including antigen delivery, bisphenol A degradation and diagnosis of Alzheimer's disease.^46,47^ Enzyme conformational changes within MIL-53NH_2_(Al) have, however, also been shown to negatively affect encapsulated enzymes, with reduced catalytic efficiency of immobilised laccase *versus* free laccase being attributed to a structural change in laccase which inhibits the active site.^47^

HKUST-1 is a highly porous, copper-based 3D MOF, with the empirical formula [Cu_3_(L9)_2_(H_2_O)]_*n*_. HKUST-1 has a face-centred cubic crystal structure which contains two comparable pores of 14 Å and one narrow pore of 10 Å.^48^ The large pores are suitable for enzyme immobilisation and are beneficial for transport of substrates involved in enzymatic reactions. Falcaro and coworkers pioneered the immobilisation of BSA into HKUST-1 *via* biomimetic mineralisation; however, the benzene-1,3,5-tricarboxylic acid ligand (L9) required dissolution in ethanol prior to the synthesis procedure, conditions that were not conducive to the maintenance of enzyme activity.^7^ In 2017 Lv and coworkers reported the biomimetic crystallisation of horseradish peroxidase (HRP) and glucose oxidase (GOx) within HKUST-1 grown on the surface of Fe_3_O_4_ nanoparticles.^49^ By varying the crystallisation sequence, they showed that HRP or GOx could be selectively immobilised on the inner or outer layer of the particle and the order of the layering of the enzyme@HKUST-1 effected both the rate of enzymatic reaction as well as the thermal stability and chemical stability of the particles. Specifically, when GOx was deposited on the outer layer and HRP in the inner layer the sequential oxidation of glucose to gluconic acid and H_2_O_2_ followed by oxidation of *o*-phenylenediamine to 2,3-diaminophenazine proceeded faster than when the enzyme layers were reversed. In contrast, the MOF composite with HRP in the outer layer and GOx in the inner layer had better stability.^49^ Similarly, laccase@HKUST-1 exhibited better stability in polar organic solvents than the free laccase enzyme, as well as good long-term stability and promising reusability for at least 10 cycles. The authors propose that copper(ii) provided an activation effect on the laccase, accelerating the electron transfer rate between active sites in the laccase enzymes.^50^

The UiO-66 family of MOFs are made up from [Zr_6_O_4_(OH)_4_]^12+^ metal clusters and aromatic dicarboxylic acid ligands L7 and L10 (Fig. 7). They all share a face-centred cubic structure and house two kinds of pores: an octahedral cage with a ∼12 Å pore, and a tetrahedral cage with a ∼7.5 Å pore; meanwhile the pore aperture size is ∼6 Å.^51^ The large pore diameters and outstanding thermal stability of UiO-66 MOFs have made them particularly appealing targets for researchers looking to synthesize protein@UiO-66s.^41^ As the formation of the [Zr_6_O_4_(OH)_4_]^12+^ cluster, an essential precursor for UiO-66 MOFs, requires high temperatures and highly acidic reaction conditions the synthesis of protein@UiO-66s has proven challenging. Several papers have however been published reporting the room-temperature synthesis of UiO-66 MOFs, but the reaction conditions often require the addition of a large amount of acid as modulator or use organic solvents such as DMF. Furthermore, these methods often give rise to poorly crystalline products incorporating defects.^39,52^ The successful immobilisation of active enzymes in UiO-66 MOFs is also controversial as many studies have demonstrated that UiO-66 MOFs and their [Zr_6_O_4_(OH)_4_]^12+^ clusters tend to hydrolyse proteins.^53^ Despite these challenges, Stoddart and coworkers report that when ligand L7 was replaced with L10 and an appropriate amino acid was included in the biomimetic crystallisation protocol the synthesis of the enzyme@UiO-66-F_4_ could proceed smoothly in water under conditions which preserve enzyme activity.^41^ Employing an alternative approach, Qu and coworkers chose to exchange the [Zr_6_O_4_(OH)_4_]^12+^ nodes with structurally related cerium nodes which can be synthesised in an aqueous solvent at ambient temperatures. Following immobilisation of GOx, uricase or xanthine oxidase into UiO-66(Ce), it was demonstrated that the respective oxidase@UiO-66(Ce) could be applied to *in vitro* detection of glucose, uric acid and xanthine. These results support each of the oxidases retaining their native activity following immobilisation within the UiO-66(Ce) framework and present a promising application for development of enzyme@MOFs as biosensors in the clinic.^54^

In addition to the established families of carboxylate-based MOFs a number of non-classified aromatic carboxylate based MOFs have been reported. These MOFs employ a wide range of metal ions and linkers and as with previous examples may display improved catalytic properties, stability and/or novel sensing or drug delivery applications.^7,45,55^

In conclusion, while a number of protein@MOFs have been identified that impart favourable properties on the immobilised protein there is still a vast research space waiting to be explored which may enable identification of MOFs with further improved properties.

### 3.2 Conformational analysis of protein@MOFs

Optimisation of a protein's conformation within a MOF provides an attractive route to enhancing the enzyme activity. When the successful pairing of proteins and MOFs is achieved enzymes including lipase,^56–58^ cytochrome *c* (CytC)^59–61^ and HRP^62^ have been shown to have better activity and stability when encased in MOFs than their unencapsulated counterparts. Analysis of the encapsulated protein is, however, challenging, with support for activated conformations being obtained through a variety of characterisation techniques, as discussed below.

**Fig. 8:**
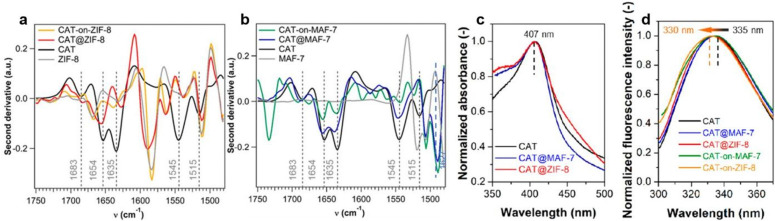
Second derivative ATR-FTIR spectra (vector normalized in the spectra region 1750–1480 cm^−1^) of; (a) ZIF-8 (gray), CAT (black), CAT@ZIF-8 (red) and CAT-on-ZIF-8 (orange) and (b) MAF-7 (gray), CAT (black), CAT@MAF-7 (blue), and CAT-on-MAF-7 (green). (c) Solid-state UV-vis spectra and (d) fluorescence spectra of free catalase (CAT, grey), encapsulated catalase (CAT@MAF-7, blue and CAT@ZIF-8, red) and surface-adsorbed catalase (CAT-on-MAF-7, green and CAT-on-ZIF-8, orange). Reproduced with permission from ref. 63.

In 2019, Sumby, Falcaro, Doonan and coworkers found that catalase (CAT) encased in hydrophobic ZIF-8 (CAT@ZIF-8) completely lost its bioactivity. In contrast, when the enzyme was encapsulated within hydrophilic MOFs, including MAF-7 and ZIF-90, the bioactivity was retained.^63^ Catalase (CAT) is an iron-heme enzyme which catalyzes the conversion of H_2_O_2_ to water and O_2_. Vibrational and optical spectroscopies were utilized to explain the observed change in catalytic activity within the different MOF structures. According to the second derivative ATR-FTIR spectra (Fig. 8a and b), free CAT showed a characteristic absorbance at 1635 cm^−1^, attributed to the intermolecular β-sheet structures. This peak was retained in CAT@MAF-7 but shifted approximately 8 cm^−1^ in CAT@ZIF-8, indicating a change in the peptide conformation. The new peak at 1627 cm^−1^ is commonly associated with intermolecular β-sheets present in protein aggregates^64^ these results therefore suggest that hydrophobic ZIF-8 induces the aggregation of immobilized CAT, ultimately leading to its loss of catalytic activity. At the same time UV-vis and fluorescence spectra revealed no changes in the heme Soret band following encapsulation in either ZIF-8 of MAF-7 (Fig. 8c and d) indicating that encapsulation did not alter the structure of the heme active site. In particular, tryptophan-derived fluorescence is known to be highly sensitive to its local environment;^65^ therefore, observation of the same absorbance and emission spectra between CAT@MOF and free CAT is a strong indicator that the MOF does not alter the enzyme active site.

In contrast, more active conformations of lipases have been reported upon immobilization with MOFs.^56–58^ Lipases catalyse the hydrolysis of ester bonds in hydrophobic compounds such as triglyceride, and generally possess an α-helical sequence which covers the active site, acting as a lid.^66^ Single crystal structures of this class of enzyme suggest that the exterior of the lid is hydrophilic whereas the interior is hydrophobic.^58,66^*In vivo* lipases are only activated when adsorbed on a water–oil interface, which opens the lid and improves the active site accessibility to substrates.^67^ Inspired by this environmentally determined behaviour, researchers have utilised hydrophobic interactions to tune the conformation of immobilized lipases. A 2.5-fold higher enzymatic activity was observed for *Thermophila lanuginosus* lipase (TL) following post-assembly infiltration of TL into the mesopores of NU-1003 functionalized with fatty acid chains (TL@NU-1003-C_22_).^57^ According to isothermal titration calorimetry (ITC) results, adsorption of TL within the NU-1003 pores is an exothermic process; however more heat was released when TL underwent infiltration into the fatty acid-modified NU-1003-C_22_ than into the parent NU-1003 framework. This suggests a stronger interaction between the protein and MOF when the fatty acids chains are present. This stronger TL-NU-1003-C_22_ binding affinity was attributed to the hydrophobic interaction between the fatty acid chain and TL, which was absent in NU-1003. Solid state NMR spectra of TL@NU-1003-C_22_ supported the correlation between an enzyme proton (7.61 ppm) and a fatty chain proton (0.33 ppm), which was not observed in TL@NU-1003. This signal could be ascribed to the spatial proximity between TL and the fatty acid. In addition, correlations between TL aliphatic protons and fatty acid carbons were also found in TL@NU-1003-C_22_, further supporting this result. Therefore, the hydrophobic interaction at the interface of the protein and functionalized MOF pores was hypothesized to tune the lipase to an open-lid configuration resulting in the observed improved bioactivity.

**Fig. 9:**
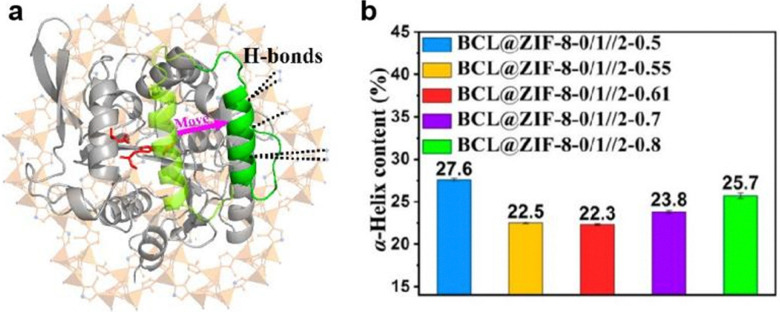
(a) Schematic representation of BCL conformation change from close-lid (pale green) to open-lid (green), induced by H-bonds between the MTV-ZIF and the α-helix lid. The active site is shown in red. (b) Calculated α-helix contents of BCL@ZIF-8-0/1//2-X based on FTIR results. Reproduced with permission from ref. 58.

In addition to the hydrophobic effect, H-bonding has also been shown to alter the lipase conformation. Recently, *Burkholderia cepacia* lipase (BCL) was encapsulated in a multivariate ZIF (MTV-ZIF) using biomimetic mineralisation.^58^ The MTV-ZIF consists of zinc(ii) and a mixture of 2-methyl imidazole (L3), 3-methyl-1,2,4-triazolate (L11) and 5-methyl-1*H*-tetrazole (L12) as its ligands, enabling the MOF hydrophilicity to be tuned by changing the ratio between linkers. The activity of encased BCL was found to vary with the MOF hydrophilicity, with the optimum activity achieved at a ligand ratio L3 : L11 : L12 of 46.3 : 46.3 : 7.4. It was proposed that for the optimized BCL@MTV-ZIF the ligand functional groups were uniquely positioned to support a change in the lipase from a closed- to an open-lid conformation (Fig. 9a), thus accounting for the improved activity. To support this hypothesis, FTIR spectroscopy was performed to study the secondary structure changes in immobilized BCL. Previous research indicates that the closed- to open-lid conformation change would decrease α-helix content and enhance activity.^66,68^ As shown in Fig. 9b, the α-helix content initially decreased before increasing as the MOF hydrophilicity increased. Moreover, the composite with the best catalytic performance was found to have the lowest degree of the α-helix suggesting that the BCL lid could be progressively opened by tuning the linker molar ratios. Similarly, the fluorescence lifetime of the different BCL@MTV-ZIFs showed a non-linear correlation with MOF hydrophilicity, with the shortest lifetime attributed to the most active MOF. This further supports the formation of an open lid structure since the lid opening would expose tryptophan to solvent, leading to fluorescence quenching and shorter lifetimes. In conclusion, increasing the MOF hydrophilicity enables formation of more H-bonds between the hydrophilic exterior of the lid and non-coordinated nitrogen atoms in the ligands, these bonding interactions support an open lid conformation which is correlated with enhanced enzymatic activity. However, beyond an optimum number of H-bonding interactions the enzyme conformation is negatively impacted, leading to activity loss.

**Fig. 10:**
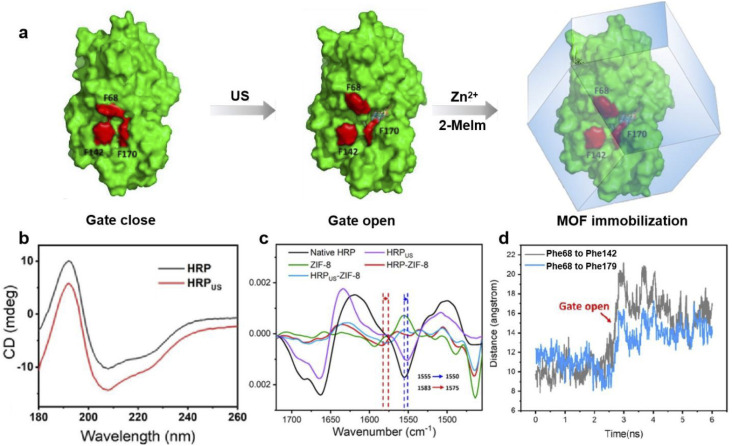
(a) Surface representations of HRP before and after US treatment; the cartoon represents the ‘locking’ of the US-activated conformation of HRP upon MOF immobilization. (b) CD spectra of HRP before and after US treatment; (c) second derivative FTIR spectra (vector normalized in the spectra region 1720–1455 cm^−1^) of HRP (black), HRP_US_ (purple), ZIF-8 (green), HRP@ZIF-8 (red), and HRP_US_@ZIF-8 (blue) (d) MD simulated distance between Phe68 and Phe142, Phe68 and Phe179 in the HRP structure during US pretreatment. Reproduced with permission from ref. 62.

Horseradish peroxidase (HRP) is another iron-heme enzyme which has been reported to become more active upon encapsulation within a MOF. In 2022 Liang and coworkers found that HRP could be activated by ultrasonication (US) and the more active but fragile conformation could be ‘locked’ within ZIF-8 upon biomimetic crystallization.^62^ The resulting HRP_US_@ZIF-8 displayed 9.3-fold higher activity than HRP immobilized without any US pretreatment. To explain this activity enhancement, the protein structure of HRP_US_ and HRP_US_@ZIF-8 were evaluated through a combination of circular dichroism (CD) spectroscopy, FTIR and computational simulation (Fig. 10). CD spectra revealed a significant increase in the α-helix, antiparallel and β-turn composition compared with the native HRP enzyme; at the same time a reduction in the parallel and random coils were observed following protein sonication. These spectral changes suggest ultrasonication alters the secondary structures of HRP. Meanwhile, the second derivative FTIR revealed that the amide I band in pristine HRP was shifted from 1555 to 1550 cm^−1^ following sonication. A similar red-shift was also observed between HRP@ZIF-8 and HRP_US_@ZIF-8, further confirming the US-induced conformation change. Molecular dynamics (MD) was also employed, and the distance between three amino acids across the active site (Phe68, Phe142, and Phe179) was measured during simulated sonication. As shown in Fig. 10d, the distance Phe68 to Phe142 and Phe68 to Phe179 increased at 2.71 ns. This step change is in agreement with a gate-closed to gate-open conformational change induced by ultrasonication. Such a conformational change would expose the heme active site promoting substrate binding, thus improving enzyme activity. In this example, although the MOF did not directly alter the HRP structure, the framework did play a key role in locking and protecting the US-activated conformation. When HRP_US_ was not encapsulated it was shown to be readily hydrolyzed resulting in a complete loss of activity. This study also demonstrated the effect of sonication on other metalloenzymes, with CAT and CytC also reported to display improved activity following sonication and immediate immobilization. This therefore appears to be a versatile route to trapping more active enzyme conformations.

Different from the aforementioned proteins, CytC is an example where MOF encapsulation has been shown to change the enzyme active site. CytC is a ferric heme oxidase which converts 2,2′-azino-bis(3-ethylbenzothiazoline-6-sulfonic acid) (ABTS) to ABTS˙^+^. Recently, CytC was infiltrated into the mesopores of NU-1000, and the resulting CytC@NU-1000 composite was reported to display twice the enzymatic activity of the free enzyme.^59^ Electron paramagnetic resonance (EPR) spectroscopy was used to study changes in CytC following immobilization. Prior to immobilisation free CytC displayed both high spin (*g* = 6) and low spin heme (*g* = 3.0, 2.2, 2.0) signals.^69^ In contrast, for CytC@NU-1000, the peaks of low spin heme decreased and a new signal emerged at *g* = 4.3, corresponding to non-heme iron or iron in a rhombic, low symmetry environment.^69^ This result suggests the MOF encapsulation altered the iron spin state at the heme active site. Using solid state UV-vis, the Soret band of CytC (410 nm) was observed to be red-shifted upon MOF immobolisation, suggesting that NU-1000 modified the microenvironment at the heme centre. The charge transfer band at 700 nm also became more intense upon MOF immobilisation, providing further support for an increase in the high spin heme.^70^ Furthermore, MD calculations were performed on CytC and the distance between iron and three peptide residues (His18, Met80, and Pro30) surrounding the heme were measured. Upon MOF immobilization, Pro30 was positioned closer to the iron, whereas the His18 and Met80 residues became more distant, which was postulated to result in enhanced substrate accessibility of the active center and thus improved enzymatic activity.

**Fig. 11:**
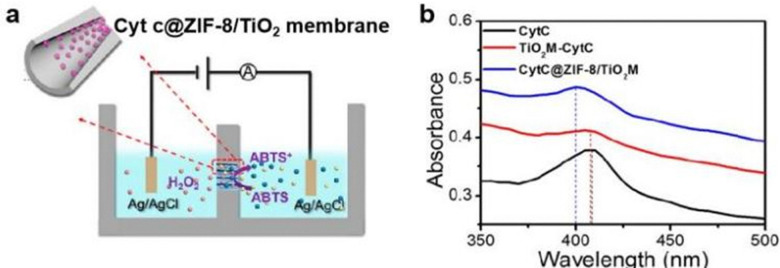
(a) Schematic illustration of CytC@ZIF-8/TiO_2_ membrane in electrolysis cell and its working mechanism. (b) UV-Vis diffuse reflectance spectra of CytC (black), TiO_2_ membrane-CytC (red), and CytC@ZIF-8/TiO_2_ membrane (blue) with high-purity BaSO_4_ employed as the background. Reproduced with permission for ref. 60.

In addition to the iron spin state, MOF encapsulation can also change the coordination sphere at the heme centre. Song and coworkers prepared a CytC@ZIF-8/TiO_2_ membrane system by functionalizing the surface of a TiO_2_ nanochannel with riboflavin sodium phosphate (RFMP), a chelating agent used to anchor zinc(ii).^60^ Then CytC and L3 were added to the nanochannel solution allowing CytC@ZIF-8 to spontaneously assemble on the TiO_2_ nanochannel walls. The resulting CytC@ZIF-8/TiO_2_ membrane was used to catalyze the oxidation of ABTS to ABTS^+^ in the presence of H_2_O_2_ in an electrolysis cell (Fig. 11a). Since ABTS^+^ ions are produced in TiO_2_ nanochannels, more transmembrane ion transport was observed when using the CytC@ZIF-8/TiO_2_ membrane compared to the non-enzyme-catalyzed electrolysis. Therefore, the encased CytC activity could be monitored by measuring the current change, and the CytC@ZIF-8/TiO_2_ membrane showed enhanced activity over the TiO_2_-immobilized enzyme (TiO_2_ membrane-CytC). To explain the improved catalysis, UV-vis was performed and compared with quantum mechanical calculations. As shown in Fig. 11b, the Soret band of CytC did not change significantly upon TiO_2_ membrane immobilization; in contrast the Soret band was blue-shifted by ∼10 nm in the CytC@ZIF-8/TiO_2_ membrane, suggesting a MOF-induced structural change had occurred at the heme centre. Quantum mechanical calculations support the hypothesis that rupture of the Fe–S bond between the iron centre and the axial Met80 would lead to a blue shift of ∼16 nm, consistent with the results obtained using UV-vis spectroscopy. The dissociation of Met80 from Fe could therefore lead to structural distortions at the heme active site, resulting in enhanced activity for the CytC@ZIF-8/TiO_2_ membrane.

ZIF-8 has also been reported to influence the spin state of encapsulated CytC. In 2024 Wang, Lv and coworkers designed a physical imprinting method in which the biomimetic crystallized CytC@ZIF-8 was calcinated to remove the protein and create a mesoporous material with pore sizes matching CytC.^61^ The enzyme was then reintroduced into the material *via* infiltration and the resulting CytC@HZIF-8_[Cyt *c*]_ showed 4.2-fold and 16.7-fold activity improvement compared to CytC@ZIF-8 and the free enzyme, respectively. The activity enhancement was attributed to protein conformational changes in CytC@HZIF-8_[Cyt *c*]_ which were supported by ssUV-vis and EPR spectral analysis. ssUV-Vis spectra indicated a 10 nm red shift of the Soret band after encapsulation, supporting a change in the heme microenvironment in the CytC@HZIF-8_[Cyt *c*]_. The EPR spectra, for the free enzyme showed high spin peaks at *g* = 6 and *g* = 4.3, whereas CytC@HZIF-8_[Cyt *c*]_ showed a prominent *g* = 2.0 signal corresponding to low spin heme, suggesting the HZIF-8 encapsulation altered the iron spin state. According to FTIR spectra, the encapsulated CytC possessed lower α-helix content than the free enzyme, implying relaxation of the protein structure in CytC@HZIF-8_[Cyt *c*]_.^71^ Moreover, 2D ^13^C–^1^H HETCOR NMR correlations were found between the enzyme protons and HZIF-8 carbon atoms, illustrating the protein-MOF interaction. In conclusion, HZIF-8 encapsulation not only changed the spin state of the heme active site but also influenced the global conformation, leading to enhanced catalytic performance.

### 3.3 Emerging techniques for analysis of protein@MOFs

As discussed in Section 3.2 different techniques have been used to probe confined protein structures; however, these techniques still suffer from limitations. For instance, EPR is only suitable for studying metalloenzymes with paramagnetic metal centres, ssUV-vis is most applicable for proteins with heme moieties, and fluorescence spectroscopy is only appropriate for tryptophan-enriched proteins. This section focusses on emerging characterization approaches which have demonstrated more general applicability for characterization of protein@MOF composites.

**Fig. 12:**
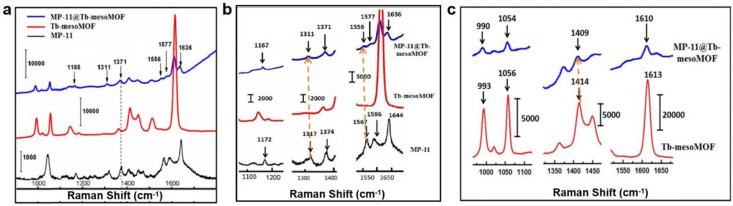
(a) Raman spectra of MP-11@Tb-mesoMOF (blue), Tb-mesoMOF (red), and 50 μM MP-11 in HEPES buffer solution (black). Enlarged regions of the spectra showing Raman band shifts (b) between MP-11 and MP-11@Tb-mesoMOF and (c) between Tb-mesoMOF and MP-11@Tb-mesoMOF. Reproduced with permission from ref. 72.

Raman spectroscopy has been used to study the interaction between immobilized MP-11, a small heme peroxidase, and the host Tb-mesoMOF.^72^ Tb-mesoMOF consists of terbium(iii) ions and a trigonal planar ligand 4,4′,4′′-*s*-triazine-2,4,6-triyltribenzoate (L13), and MP-11 was immobilized within its mesopores following the MOF synthesis. Following protein uptake, no leaching of MP-11 from the MOF was observed. To explain this phenomenon, the Raman spectra of free enzyme, pure MOF and MP-11@Tb-mesoMOF were compared. As shown in Fig. 12, the protein peak at 1371 cm^−1^ corresponding to ν_4_(C–N) of the heme was retained in MP-11@Tb-mesoMOF but not observed in the pure MOF, suggesting the successful protein adsorption in MOF pores. In the magnified spectra (Fig. 12b), the enzyme peaks at 1596 and 1644 cm^−1^ were red shifted upon MOF immobilization. According to previous reports, these shifts are related to the disruption of the protein dimer or oligomer structures.^73^ Instead a single MP-11 monomer is believed to be accommodated per pore. Four additional peaks (1172, 1317, 1374, and 1567 cm^−1^) corresponding to heme vibrational modes were also reported to be red shifted, suggesting that MP-11 interacts with the MOF *via* its heme moiety. In the pure MOF spectra (Fig. 12c), peaks corresponding to the C=C stretching within the ligand benzene ring (1613 cm^−1^) and vibration bands of triazine (993, 1414 cm^−1^) were also observed to be red-shifted upon protein adsorption, suggesting that Tb-mesoMOF interacts with the enzyme through the aromatic rings in the TATB ligand. Notably, the triazine vibration peak (1414 cm^−1^), and the C–H and C=C peaks of the protein heme (1317, 1567 cm^−1^) saw the largest red shift upon MP-11@Tb-mesoMOF formation, indicating a strong interaction between the enzyme heme and triazine in the ligand. These results suggest the heme active site in MP-11 interacts with the triazine and benzene rings in the ligand through π–π interactions and these weak interactions prevent leaching of the adsorbed protein. In conclusion, Raman spectroscopy is a versatile approach for studying the interaction between a MOF and confined protein, and the technique has broad applicability so it could be utilised in analysis of most protein@MOF composites.

**Fig. 13:**
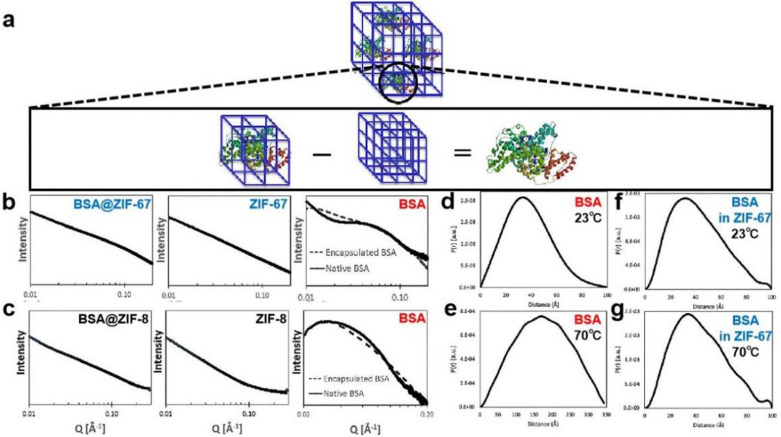
(a) Schematic representation showing scaled subtraction of pure MOF spectra from enzyme@MOF spectra to reveal the encapsulated BSA structure. log–log scale SAXS spectra of (b) BSA@ZIF-67 (left), pure ZIF-67 (middle), native or encapsulated BSA in ZIF-67 (right); and (c) BSA@ZIF-8 (left), pure ZIF-8 (middle), native or encapsulated BSA in ZIF-8 (right). PDDF plots of (d) 4 mg mL^−1^ native BSA in HEPES buffer at 23 °C; (e) 4 mg mL^−1^ native BSA in HEPES buffer at 70 °C; (f) calculated spectra of BSA encapsulated in ZIF-67 at 23 °C; and (g) calculated spectra of BSA encapsulated in ZIF-67 at 70 °C. Reproduced with permission from ref. 74.

Recently, small angle X-ray scattering (SAXS) has also been employed to study the structure of a protein encapsulated within a MOF. Prausnitz, Walton and coworkers developed a subtraction method in which the scaled SAXS spectra of the pure MOF was subtracted from the BSA@MOF spectra to reveal the conformation of encapsulated BSA (Fig. 13).^74^ As shown in Fig. 13b and c, the plots for BSA confined in ZIF-67 and ZIF-8 are similar to the native protein spectra. However, slight differences were observed in the *q* range of 0.01–0.03 Å^−1^, these differences are attributed to inhibited protein aggregation upon MOF encapsulation. The SAXS spectra were then Fourier-transformed to get the pair distance distribution functions (PDDF), which allow a more quantitative study of the protein size and geometry. According to the PDDF plots, free and encased BSA displayed similar bell-shaped distributions at room temperature, suggesting both native and confined proteins are in a globular, well-folded conformation. However, upon *in situ* heating at 70 °C, the PDDF plot of free BSA significantly broadened, and the protein radius of gyration (*R*_g_), determined from PDDF, dramatically increased from 29.53 Å at 23 °C to 133.0 Å at 70 °C (Fig. 13d and e). This result suggests the unfolding of BSA at elevated temperature and the loss of its spherical conformation. In contrast, BSA encapsulated in ZIF-67 (Fig. 13f and g) showed no obvious change in the curves of the *R*_g_ values at 23 °C (32.21 Å) and at 70 °C (33.34 Å), indicating the encased protein retained its original structure. Therefore, the confined space in the MOF prevents the protein from unfolding, leading to enhanced thermal stability. In conclusion, this work introduces a new method to study immobilized protein@MOF conformations, employing *in situ* heating to determine thermal stabilisation arising from MOF encapsulation.

**Fig. 14:**
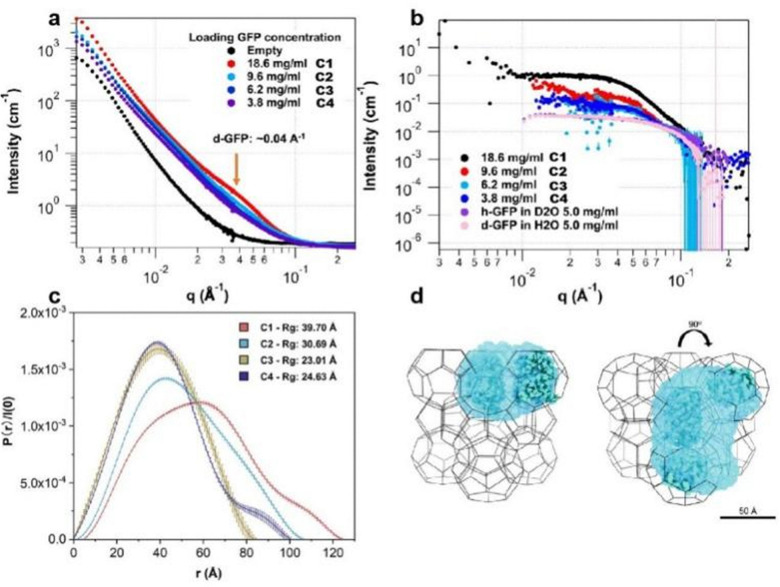
(a) SANS spectra of dGFP@MOF-919 with different protein loading content at the contrast matching point 50% D_2_O. (b) Calculated spectra of dGFP confined in MOF-919 after the subtraction of MOF scattering. (c) Pair distance distribution functions (*P*(*r*)) of dGFP@MOF-919 with different protein loading content (C1–C4). (d) *Ab initio* structure reconstruction of C2 in the structure of MOF-919. The blue-shaded region is reconstructed using the scattering profile of C2, and the GFP atomic structure is overlaid as blue balls for comparison. Reproduced with permission from ref. 75.

Similarly, small angle neutron scattering (SANS) was also used to study the protein conformation and spatial arrangement within a MOF.^75^ Using a similar approach to that taken by Prausnitz, Walton and coworkers with the SAX spectra, the SANS spectra of the pure MOF (MOF-919) were subtracted from GFP@MOF-919 spectra to obtain the scattering profile of the encapsulated green fluorescent protein (GFP). SANS, however, has an advantage over SAX due to its contrast variation function. Since the neutron scattering length density (NSLD) of protons (^1^H, −3.742 fm) and deuterions (^2^H or D, 6.675 fm) have opposite signs, a mixed solvent of H_2_O and D_2_O can be optimized to selectively match the NSLD of MOF networks. At this optimized composition, the scattering profile of the MOF is similar to the solvent at the contrast matching point, and thus can be easily subtracted from the protein@MOF spectra to highlight the confined protein scattering. For MOF-919, the contrast matching point was measured to be 50% D_2_O by volume, and this ratio was used for all protein@MOF analyses. On the other hand, GFP was deuterated (d-GFP) before MOF adsorption in order to increase the scattering contrast between MOF backbone and immobilized protein. SANS spectra were measured for d-GFP@MOF-919 with different protein loading contents (18.6 to 3.8 mg mL^−1^, termed as C1–C4), and the intensity increased dramatically at higher protein concentration due to the increased deuterium in the sample (Fig. 14a). For C1, a hump was observed at 0.04 Å^−1^, which is a signature of d-GFP. The MOF-919 spectrum was then subtracted from all the d-GFP@MOF-919 curves to get the scattering patterns for encapsulated d-GFP (Fig. 14b), which were further transformed to pair distance distribution functions (*P*(*r*)) to determine the *R*_g_ of the confined protein. As shown in Fig. 14c, C3 and C4 possess similar distributions and the *R*_g_ values are analogous to free d-GFP in H_2_O (22.31 Å). In contrast, with higher protein loading, C1 and C2 display broader distributions and much larger *R*_g_ values, suggesting the formation of oligomers in the MOF. This is further confirmed with a 3D reconstruction of GFP@MOF-919 (Fig. 14d), which indicates that GF molecules are adsorbed in adjacent mesopores, and the apertures in cavities allow them to interact with each other to form these protein assemblies. This work for the first time demonstrates that the protein arrangement in a MOF can be directly visualized. The contrast matching method combined with protein deuteration enable a strong protein signal to be isolated from the protein@MOF composite, highlighting the unique potential of SANS for characterization of protein@MOF composites.

In conclusion, a number of lab-based and specialist characterization techniques have been employed to better understand the positioning and conformational changes of proteins following their immobilization within MOFs. Some of these techniques are dependent on the inherent properties of the protein whilst others are more generally applicable. In all instances, however, understanding of the complexed protein@MOF structures benefits from the input of multiple different characterization techniques.

## Conclusions

In recent years the number of reports for both MOCs and MOFs encapsulating peptides and proteins has increased significantly as the potential applications of these MOMs is being recognised. The well-defined and often modular structures of MOMs allow for systematic variation of the internal cavity spaces and thus a near infinite array of encapsulation environments for biomolecules. Moreover, the facile self-assembly approaches utilised in the generation of these materials alongside the batch-to-batch reproducibility and atomic level characterisation of MOMs present opportunities for developing an in depth understanding of how the MOM structure is directly altering the performance and conformation of the protein or peptide. In particular, the discrete, soluble nature of MOCs has enabled significant insight into peptide@MOC binding, with clear examples demonstrating microenvironment enforced conformational changes akin to behaviour observed in biological chaperone molecules. Limitations associated with the assembly of MOCs, specifically the synthesis of water-soluble MOCs with large interior cavities, have, however, limited the number of examples of MOCs that have been tested for peptide binding, and, to date, encapsulation of a catalytically active protein has yet to be reported.

In contrast, protein@MOF studies have focussed on the activity of enzymes following immobilisation as characterisation of the protein within the MOF remains a significant challenge. Recent reports demonstrate the value of specialist spectroscopic techniques in addressing this characterisation challenge; however, the ability to use common lab-based techniques, such as solution state NMR and single crystal X-ray diffraction, as utilised for the peptide@MOC systems would enable for more rapid and conclusive data to be presented with regard to conformational changes of proteins within MOFs.

Future work should therefore look to better integrate results from MOC and MOF studies enabling understanding of conformational changes upon encapsulation of peptides@MOCs to be incorporated into the design of protein@MOF systems. At the same time, where good or unexpected enzymatic behaviour is observed for protein@MOF systems the development of MOC based analogues should be considered to enable a better understanding of the conformational changes responsible for the enzymatic behaviour.

## Author contributions

Conceptualization, formal analysis, resources, review & editing, J. D. W., T. Z., X. W. and I. A. R.; Methodology, investigation, visualization, J. D. W., T. Z., and X. W.; writing – original draft, J. D. W., T. Z. and X. W.; supervision, funding acquisition, I. A. R.

## Data availability

No primary research results, software or code have been included and no new data were generated or analysed as part of this review.

## Conflicts of interest

There are no conflicts to declare.
